# [Retracted] MicroRNA-152 inhibits tumor cell growth by directly targeting RTKN in hepatocellular carcinoma

**DOI:** 10.3892/or.2025.8965

**Published:** 2025-08-04

**Authors:** Jiejing Zhou, Yanjun Zhang, Yuhong Qi, Dequan Yu, Qiuju Shao, Jun Liang

Oncol Rep 37: 1227–1234, 2017; DOI: 10.3892/or.2016.5290

Following the publication of the above paper, it was drawn to the Editor's attention by a concerned reader that, regarding the flow cytometric plots shown in Figs. 2D and 4C, several of the panels appeared to show similar groupings of dots, which would not have been anticipated if these experiments had been performed discretely under different experimental conditions, suggesting a fundamental flaw either in the way in which these experiments were performed, or in how the results were outputted.

The Editor of *Oncology Reports* has decided that this paper should be retracted from the Journal on account of a lack of confidence in the presented data. The authors were asked for an explanation to account for these concerns, but the Editorial Office did not receive a reply. The Editor apologizes to the readership for any inconvenience caused.

